# Meiotic drive in chronic lymphocytic leukemia compared with other malignant blood disorders

**DOI:** 10.1038/s41598-022-09602-1

**Published:** 2022-04-12

**Authors:** Viggo Jønsson, Haneef Awan, Neil Deaton Jones, Tom Børge Johannesen, Klaus Thøgersen, Bjarni á Steig, Gudrid Andorsdottir, Geir Erland Tjønnfjord

**Affiliations:** 1 Department of Hematology, Oslo University Hospital, and Institute of Clinical Medicine, University of Oslo, Nydalen, P.O. box 4950, 0424 Oslo, Norway; 2 Center for Information Technology Service (USIT), Oslo University Hospital, and Institute of Clinical Medicine, University of Oslo, Blindern, P.O. box 1059, 0316 Oslo, Norway; 3 Department of Computer Science, University of Copenhagen, Universitetsparken5, Building B, 2100 Copenhagen, Denmark; 4 Norwegian Cancer Registry, Ullernchausseen 64, 0379 Oslo, Norway; 5 Technology, Product Development, Medical Device Development (TPU Ltd.), Mølledamsvej 10, 3460 Birkerød, Denmark; 6 National Hospital of the Faroe Islands, Medical Department, J.C. Svaboe gøta 2, F 100 Torshavn, Faroe Islands; 7 Genetic Biobank of the Faroe Islands, J.C. Svaboe gøta 43, 100 Torshavn, Faroe Islands; 8 Department of Hematology, Oslo University Hospital and K.G, Jebsen Center for B-Cell Malignancies and Institute of Clinical Medicine, University of Oslo, Nydalen, P.O. box 4950, 0424 Oslo, Norway

**Keywords:** Cancer genetics, Cancer genetics

## Abstract

The heredity of the malignant blood disorders, leukemias, lymphomas and myeloma, has so far been largely unknown. The present study comprises genealogical investigations of one hundred and twelve Scandinavian families with unrelated parents and two or more cases of malignant blood disease. For comparison, one large family with related family members and three hundred and forty-one cases of malignant blood disease from the Faroese population was included. The inheritance is non-Mendelian, a combination of genomic parental imprinting and feto-maternal microchimerism. There is significantly more segregation in maternal than in paternal lines, predominance of mother-daughter combinations in maternal lines, and father-son combinations in paternal lines. Chronic lymphocytic leukemia is the most frequent diagnosis in the family material, and chronic lymphocytic leukemia has a transgenerational segregation that is unique in that inheritance of susceptibility to chronic lymphocytic leukemia is predominant in males of paternal lines. Male offspring with chronic lymphocytic leukemia in paternal lines have a birth-order effect, which is manifest by the fact that there are significantly more male patients late in the sibling line. In addition, there is contravariation in chronic lymphocytic leukemia, i.e. lower occurrence than expected in relation to other diagnoses, interpreted in such a way that chronic lymphocytic leukemia remains isolated in the pedigree in relation to other diagnoses of malignant blood disease. Another non-Mendelian function appears in the form of anticipation, i.e. increased intensity of malignancy down through the generations and a lower age at onset of disease than otherwise seen in cases from the Cancer Registers, in acute lymphoblastic leukemia, for example. It is discussed that this non-Mendelian segregation seems to spread the susceptibility genes depending on the gender of the parents and not equally to all children in the sibling line, with some remaining unaffected by susceptibility i.e. "healthy and unaffected", due to a birth order effect. In addition, anticipation is regarded as a non-Mendelian mechanism that can amplify, «preserve» these vital susceptibility genes in the family. Perhaps this segregation also results in a sorting of the susceptibility, as the percentage of follicular lymphoma and diffuse large B-cell lymphoma is lower in the family material than in an unselected material. Although leukemias, lymphomas and myelomas are potentially fatal diseases, this non-Mendelian distribution and amplification hardly play any quantitative role in the survival of Homo sapiens, because these diseases mostly occur after fertile age.

## Introduction

Non-Mendelian inheritance (meiotic drive) of susceptibility to mutation in the hematopoietic stem cells is the etiology of malignant blood disorders (MBD)^1^. Chronic lymphocytic leukemia (CLL), the most common leukemia in the Western World, is a subset of MBD^1,2^. Together with other types of malignant lymphoproliferative disorders (LPD) such as acute lymphoblastic leukemia, other subsets of lymphoid leukemia, malignant lymphomas including Hodgkin lymphoma, myeloma and the myeloproliferative disorders (MPD), these all depend on a monoclonal expansion of blood cells derived from a mutated hematopoietic stem cell^1–3^. Each disorder has its own cytogenetic profile^4–10^, forming an inherited polygenetic model of disease susceptibility^11–13^ with an evident familial occurrence^14–21^.

**Figure 1:**
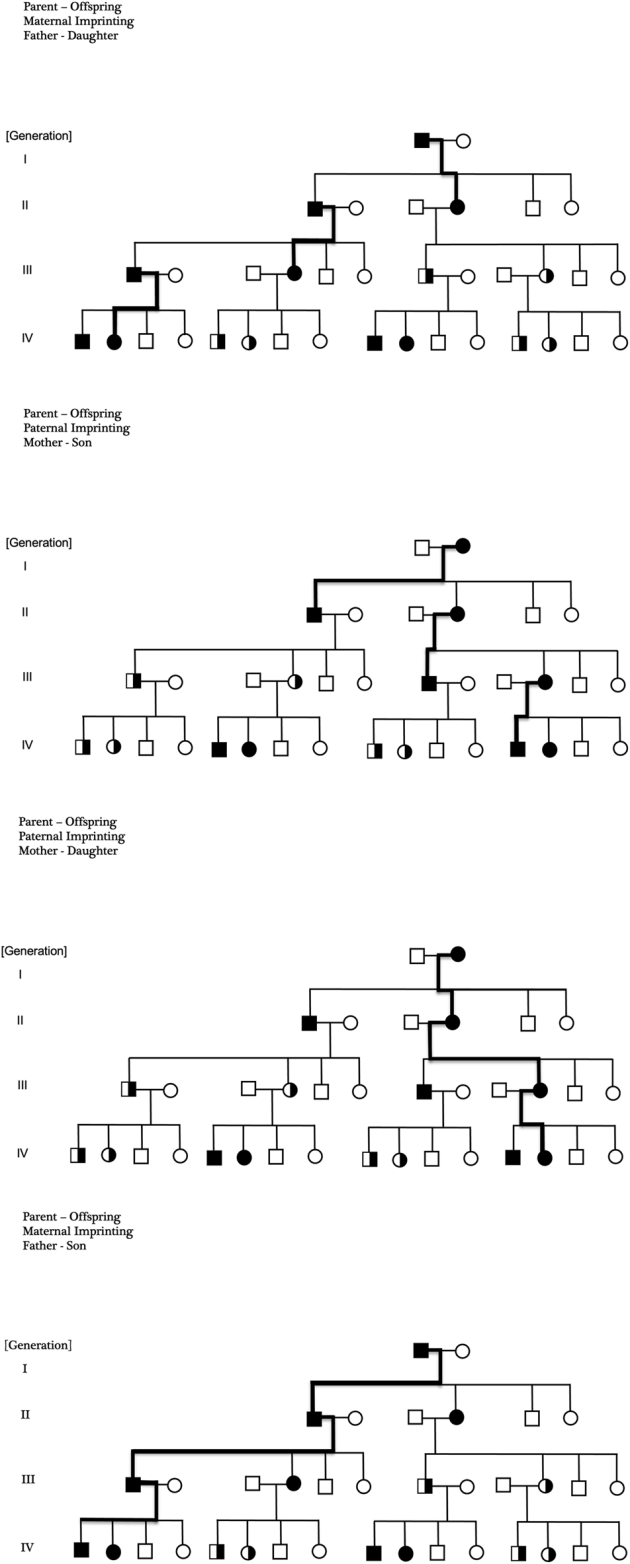
Parental genomic imprinting denotes a transgenerational segregation that depends on the gender of the parents. One allele is transcriptionally inactivated (imprinted, silenced) while the other allele remains active. At each transgenerational passage, the imprint erases and then it regenerates making imprinting a lifelong mechanism. Transgenerational pathways specific to men and women in paternal- and maternal lines are shown in bold. Signature: Square, male. Circle, female. Black, affected. White, unaffected. Black and white, carrier. Black bold line, main pathway of segregation.

It has been difficult to get a comprehensive overview of the inheritance process in spite of an intensive search for decades. We have proposed a segregation pattern of MBD based on the interaction of parental genomic imprinting^22–25^ and fetomaternal microchimerism^26–28^ (Fig. 1). That model^29^ gathers and explains the details known about the inheritance pathway in general, but CLL stands out. Genealogical studies of pedigrees from affected families show that CLL seems to have a meiotic drive of its own paths with a unique distribution of affected ancestors to a CLL proband^29^. Only CLL has a demonstrable birth order effect (Table 1) and clinically, CLL-males and females have a different course of disease^30,31^. Female patients usually have a better crude survival than males, a higher age at onset of disease, and a better dose–response to chemotherapy^30,31^. These clinical differences between male and female patients sometimes occur in non-Hodgkin lymphomas (NHL)^32,33^, but not as clear as in CLL. Furthermore, we have recently assessed the distribution in the pedigree of MBD-affected ancestors to a proband with MBD^29^.

**Table 1:** Proband crude (PC) of familial malignant blood disorders.

Lymphoproliferative disorders—LPD ICD-10 Code	Norwegian and Danish families				The Faroese family			
	Total	(males, females)	%	Male, Female Ratio	Total	(males, females)	%	Male, Female Ratio
Chronic lymphocytic leukemia CLL C91.1	181	(98, 83)	65.6	1.2	50	(29, 21)	22.8	1.4
Acute lymphoblastic leukemia ALL C91.0	4	(2, 2)	1.4	1.0	16	(10, 6)	7.3	1.7
Other Leukemias	14	(7, 7)	5.1	1.0	5	(3, 2)	2.3	1.5
Multiple myeloma MM C90	10	(6, 4)	3.6	1.5	50	(31, 19)	22.8	1.6
Monoclonal gammopathy MGUS D47.2	2	(1, 1)	0.7	1.0	0			
Hodgkin lymphoma HL C81.1	11	(8, 3)	4.0	2.7	19	(12, 7)	8.7	1.7
Diffuse large B-cell lymphoma DLBCL C83.3	16	(9, 7)	5.8	1.3	34	(21, 13)	15.6	1.6
Follicular lymphoma FL C82	19	(13, 6)	6.9	2.2	14	(9, 5)	6.4	1.8
Other lymphomas	19	(11, 8)	6.8	1.4	31	(16, 15)	14.1	1.1
Lymphoproliferative disease LPD total	276	(155, 121)	100	1.3	219	(131, 88)	100	1.5
Age at onset of disease years (median)	67	(65, 69)			61	(58, 64)		
Birth order effect	CLL males, paternally				No			
Parent offspring pairs	126 pairs				2 pairs			
Siblings	42 pairs				1 pair			
**Myeloproliferative disorders—MPD**								
Acute myeloid leukemia AML C92.0 and C92.2-9	9	(6, 3)	37.6	2	44	(28, 16)	50.6	1.8
Chronic myeloid leukemia CML C92.1	3	(1, 2)	12.5	0.5	14	(9, 5)	16.1	1.8
Other MPD	12	(6, 6)	50.0	1.0	29	(15, 14)	33.3	1.1
Total MPD	24	(13, 11)	100	1.2	87	(52, 35)	100	1.5
Age at onset of disease								
Years (median)	63	(59, 68)			54	(49, 60)		
Birth order effect	No				No			
Parent offspring pairs	12 pairs				0 pairs			
Siblings	1 pair				0 pairs			
**Other malignant blood disorders**								
Leukemia, uncertain L NOS C95.9	1	(1, 0)			7	(2, 5)		
Malignant histiocytosis MH C96.1	0				2	(0, 2)		

NOS, not otherwise specified.

Supplementary studies of this previously used family material (Table 1) shows differences in paternal and maternal affiliation by inheritance of MBD (Table 2) and a pattern of segregation with co- and contravariation (Tables 3 and 4) and here CLL stands out.

**Table 2:** Paternal (PA) and Maternal (MA) affiliation of affected parent – offspring in pairs from families in Norway and Denmark with unrelated parents.

CLL pairs	PA			MA		
	Total	Males	Females	Total	Males	Females
CLL parents	16	16	0	26	0	26
CLL children	16	11	5	26	11	15
CLL grandparents	1	1	0	13	7	6
CLL grandchildren	1	1	0	13	8	5
Total CLL parents and grandparents	17	17	0	39	7	32
Total CLL children and grandchildren	17	12	5	39	19	20
Per cent children and grandchildren	100	71	29	100	49	51
**Mixed pairs CLL and nonCLL**						
CLL parents	7	7	0	11	0	11
NonCLL children	7	2	5	11	5	6
CLL grandparents	0			6	2	4
NonCLL grandchildren	0			6	2	4
NonCLL parents	9	9	0	9	0	9
CLL children	9	3	6	9	5	4
NonCLL grandparents	8	8	0	7	4	3
CLL grandchildren	8	4	4	7	5	2
Total CLL parents and grandparents	7	7	0	17	2	15
Total CLL children and grandchildren	17	7	10	16	10	6
Per cent children and grandchildren	100	41	59	100	63	38
Total nonCLL parents and grandparents	17	17	0	16	4	12
Total nonCLL children and grandchildren	7	2	5	17	7	10
Per cent children and grandchildren	100	29	71	100	41	59
**NonCLL pairs (nonCLL–nonCLL)**						
NonCLL parents	6	6	0	5	0	5
NonCLL children	6	4	2	5	3	2
NonCLL grandparents	3	3	0	11	5	6
NonCLL grandchildren	3	3	0	11	2	9
Total nonCLL parents and grandparents	9	9	0	16	5	11
Total nonCLL children and grandchildren	9	7	2	16	5	11
Per cent children and grandchildren	100	78	22	100	31	69

**Table 3:** Co- and contravariation of affected relatives to probands crude with familial malignant blood disorders (the Faroe Islands).

Probands crude (PC)	Affected relatives (AR)							
ICD-10 codes	Observed (OBS) versus expected (EXP), total number (males, females)							
	Increase, covariation MA			Increase, covariation			Decrease, contravariation	
HL C81	OBS EXP	HL: 17 (10, 7) HL: 11 (7, 4) P < 0.05						
FL C82	OBS EXP	MM: 22 (14,8) MM: 15 (9, 6) P < 0.05						
DLBCL C88.3	OBS EXP	DLBCL: 37 (14, 23) DLBCL: 28 (17, 11) P < 0.001					OBS EXP	CLL: 33 (11, 22) CLL: 41 (24, 17) P < 0.01
NHL NOS C85.9	OBS EXP	HL: 19 (12, 7) HL: 12 (8, 4) P < 0.01						
MM C90	OBS EXP	FL: 27 (15, 12) FL: 20 (13, 7) P < 0.05		OBS EXP	MM: 88 (50, 38) MM: 71 (44, 27) P < 0.01		OBS EXP	CLL: 58 (40, 18) CLL: 71 (41, 30) P < .05
	OBS EXP	ALL: 27 (11, 16) ALL: 23 (14, 9) P < 0.05		OBS EXP	CML: 33 (18, 15) CML: 22 (14, 8) P < 0.01			
	OBS EXP	MF: 29 (16, 13) MF: 12 (6, 6) P < 0.001						
AML C92.0	OBS EXP	AML: 78 (45, 33) AML: 60 (38, 22) P < 0.01		OBS EXP	DLBCL: 47 (25, 22) DLBCL: 33 (20, 13) P < 0.01		OBS EXP	CLL: 28 (17, 11) CLL: 48 (28, 20) P < 0.001
	OBS EXP	MM: 73 (41, 32) MM: 48 (30, 18) P < 0.05						
CML C92.1	OBS EXP	AML: 37 (22, 15) AML: 27 (17, 10) P < 0.05						

Diagnosis abbreviations: Cf. TABLE 1.

OBS: Number of patients observed.

EXP: Calculated number of patients using prevalence given in Table 1.

Covariation: OBS > EXP.

Contravariation: OBS < EXP.

**Table 4:** Paternal (PA) and Maternal (MA) affiliation of Affected relatives (AR) in co- and contravariation (shown in Table 3), the Faroe Island.

**Covariation**			
AR in Pc-AR pairs, lymphoproliferative disorders (LPD) shown in Table 3 with covariation			
	**AR PA**	**AR MA**	**AR Total**
1. Total (males , females)	86 (43, 43)	151 (83, 68)	237 (126,111)
%	36 (34, 39)	64 (66, 61)	100
2.For comparison: AR in all PcLPD-AR pairs of the material			
	**AR PA**	**AR MA**	**AR Total**
2. Total (males, females)	593 (349, 244)	563 (329, 234)	1156 (678, 478)
%	51 (51, 51)	49 (49, 49)	100
3. Difference between 1. and 2. (%):			
	**AR PA**	**AR MA**	
	minus 15 ( minus 17, minus 12)	15(17, 12)	
**Covariation**			
AR in Pc-AR pairs, myeloproliferative disorders (MPD) and mixed LPD and MPD shown in Table 3 with covariation:			
	**AR PA**	**AR MA**	**AR Total**
4. Total (males , females)	110 (68 ,42)	187 (99, 88)	297 (167,130)
%	37 (41, 32)	63 (59, 68)	100
5. For comparison: AR in all PcMPD-AR pairs of the material			
	**AR PA**	**AR MA**	**AR Total**
5. Total (males, females)	398 (240, 158)	366 (213, 153)	764 (453, 311)
%	52 (53, 51)	48 (47, 49)	100
6. Difference between 4. and 5. (%):			
	**AR PA**	**AR MA**	
	minus 15 ( minus 12, minus 19)	15(12, 19)	
**Contravariation**			
AR in PcLPD-ARCLL pairs and from PcMPD-ARCLLpairs, myeloproliferative disorders (MPD) and mixed LPD and MPD shown in Table 3 with contravariation:			
	**AR PA**	**AR MA**	**AR Total**
7. Total (males , females)	65 (42 ,23)	54 (26, 28)	119 (68,51)
%	55 (62, 45)	45 (38, 55)	100
8. For comparison: AR in all PcMPD-AR pairs of the material			
	**AR PA**	**AR MA**	**AR Total**
8. Total (males, females)	116 (72, 44)	159 (88, 71)	275 (160, 115)
%	42 (45, 38)	58 (55, 62)	100
9. Difference between 7. and 8. (%):			
	**AR PA**	**AR MA**	
	13 (17, 7)	minus 13( minus 17, minus 7)	

The purpose of the present paper is to describe the characteristics of the transgenerational segregation of susceptibility to MBD and to outline the modifications of the general segregation model (Fig. 1) that CLL causes.

## Material and methods

The material consists of two types of MBD-families: 1) Families with unrelated parents (112 families and 301 cases of MBD) from Norway and Denmark. 2) One big family with related parents (315 cases of MBD from the endemic, consanguineous Faroese population). Percentages of MBD (i.e., rates, prevalence or frequencies) are in use because no data from the Faroe Islands is available for calculations of incidences. Official data from the Cancer Registries or other public health institutions regarding familial MBD do not exist either. Parts of this joint database were in use for genealogical investigations elsewhere^29,34,35^.

### Birth order effect

Birth order effect (BOE), which is a rank order by age of the affected sibs in a sib ship was systematically assessed in the families by means of the Haldane & Smith’s method^36^. This method is based on a comparison of the sum of rank numbers in the test sample with a theoretical rank sum as if no BOE exists, expressed as the 95% confidence interval (CI 95%). For control, the Wilcoxon signed rank test was used, comparing the sum of older (positive scores) and younger (negative scores) of healthy sibs to the affected sib in the sib ship (significance P < 0.05). Statistical analysis was programmed using language R version 3.3 (https://www.r-project.org/).

### Anticipation

That is increased malignancy down through the generations and a lower age of onset of disease. The number of patients within each diagnosis in the Norwegian and Danish families has been compared with the number of patients with the same diagnosis in the Cancer Registries of Norway and Denmark (chi-squared test, significance P < 0.05). Age at onset of disease was also included in this comparison.

### The Norwegian and Danish MBD-families

One hundred and twelve families have been found in our hematologic out-patient clinics by asking new patients about other family members with possible MBD. Families with two or more cases of MBD were included. There were 276 cases of LPD (lymphoproliferative disorders), and 24 cases of MPD (myeloproliferative disorders), and one case of Leukemia NOS (Not Otherwise Specified), giving 2.7 patients per family.

The included persons were all of Scandinavian origin, there were no twins, and none have had unrelated parents. The observation period was largely the same as for the Faroese material. The oldest patient is a female with CLL, born in 1864.

All patients were cross-checked with the National Cancer Registries, and all members of the family, the healthy persons as well, were checked with the Civil Person Registry. In case of doubt, we checked with church books, midwives protocols, and transcripts from alimony judgements. All medical files were examined, and all diagnoses were cross-checked with the SNOMED registration of the pathologists^37^. The ICD-10 nomenclature was used for a standardization of the diagnoses from different periods with different taxonomic systems^38^.

### Parental affiliation

Each case of MBD in the family tree (designated proband crude, Pc) was associated with its affected relatives (ARs) and used for a systematic registration of familial MBD. Strictly vertical pairs of affected Pc – affected AR (viz. affected parent – offspring pairs) were selected for an estimation of the parental affiliation to ensure that only patients with a position in the pedigree that allow direct, transgenerational transfer of susceptibility from parent to child were involved (Table 2). Horizontal pairs such as concordance of two affected siblings are not included in the table. So-called oblique combinations of pairs, such as uncle, aunt–nephew, cousin were not included, neither are so-called oblique pairs (affected uncle or aunt–affected nephew or cousin).

### Co- and contravariation

Covariation indicates a greater number of observed ARs (OBS) to a Pc than expected (EXP), while contravariation indicates a smaller number of OBS than EXP (chi-squared test).

Pairs of affected Pc –affected parent were sorted by the diagnoses CLL or nonCLL (all other diagnoses of MBD than CLL). This makes it possible to compare groups of pairs with AR CLL and AR nonCLL (Table 2).

Legal permissions to do the study: cf. Acknowledgements.

### The Faroese MBD-family

As of December 2011, 341 cases of MBD have been recorded in the Faroese Diagnostic Registry in Torshavn, the capital of the Faroese Islands. During the years after 2011, all patients were cross-checked and patients with a delayed diagnosis were included. Three patients were excluded because the medical records have been lost, and 23 failed inclusions because their position in the family tree was uncertain (e.g. extra matrimonial relationship). Thus, 315 MBD patients, designated proband crude (Pc), were included since the first person (a female with CLL, born in 1884).

The Faroese people are descended from the Vikings^39^. Today, there are nearly 53.000 people from a few thousand founders who survived the plague during the Black Death about 20 generations ago around 1350^40^.The population was under the influence of isolation and consanguinity right up to a period after WW II^40^. Foreign settlers (on an average of one or two per year one hundred years ago) were mainly Scandinavian sailors and priests. The Coefficient of Inbreeding has never been calculated^41^. The Faroese parity is one of the highest in Europa (7.4 in 1900 and 2.7 today)^40^. Until 1870–80, childbed fever was a common cause of maternal death, and many men were married several times. Then, after the introduction of high-sea fishing from small boats, many men drowned and many women married again.

The course of disease, symptoms, the medical record and the pathologist’s SNOMED diagnoses^37^ were cross-checked. All included diagnoses were grouped according to the ICD-10 diagnostic system^38^, and reassessed according to the diagnostic criteria valid at the time of diagnosis. In 45 cases, NOS (not otherwise specified)-diagnoses were used: (25 cases of uncertain subtype of NHL,15 cases with uncertain distinction between myelodysplasia (MDS) and other subsets of MPD, and in five cases of lymphoid leukemia in elderly desolate patients who were not further investigated. There were no twins among the patients. BOE and anticipation were included in the screening.

### Pedigree and parental affiliation

One united pedigree showing all affected and unaffected family members was provided by the Faroese National Civil Registry. Each patient was appointed to be proband crude (Pc), and a systematic registration of the affected relatives (ARs) in up to 5 generations before each Pc allowed an estimation of the parental affiliation of each AR related to Pc. Up to five healthy family members between Pc and AR in the Faroese pedigree defined the association between Pc and AR.

Since a given patient in the generations before Pc may act as AR to several Pcs at the same time, the total number of ARs is higher than the actual number of patients with MBD.

### Co- and contravariation

The calculation of EXP for this comparison rests on the prevalence given in Table 1. In HL for example: HL represents 8.7% of all Faroese LPD (Table 1). According to our joint database, 121 AR LPD (68 males and 53 females) are related to HL so that the expected number of AR HL is 8.7% of 121 = 10.53 $\sim$ 11 HL patients (males 10.53 × 12/19 = 6.65 $\sim$ 7 patients; females 10.53 × 7/19 = 3.88 $\sim$ 4 patients). Table 3 shows all detected significant discrepancies between OBS and EXP.

Legal permissions to do the study: cf. Acknowledgements.

### Anonymity

To ensure anonymity at publication, the patients have numbers without names or initials, and without data on gender, age, or place of birth so that no person can be recognized from the outside.

### Informed consent

All patients older than 18 years got oral and written information about the purpose of the study, that participation was voluntary and could be interrupted at any time. It was stated that all data was confidential and made anonymous, and that the investigation was approved by the Scientific Ethical Committees, and the National Data Registry Agencies. Included patients accepted their participation by completing a signed questionnaire. Each patient provided informed consent to participate in the study, thus informed consent was taken from all patients. Patients under the age of 18 years were included with informed consent from a parent or a legal guardian.

### This study was approved by ethics committee

Of the Ministry of Health and Social Service, Government of the Faroe Islands, and (for Norway) the Norwegian Research Ethical Committees, that includes the Data Inspectorate, the Social and Health Directorate and the Regional Committee for Medical and Health Research Ethics, South-East Norway. For Denmark the Royal Danish National Archives, comprising the Provincial Archives of Zealand, the Danish Data Protection Office, the Danish Scientific-Ethical Committees and the Danish Board of Health.

## Results

**The diagnoses** of the two groups (continental families from Norway and Denmark with unrelated parents; and the endemic, Faroese family with related parents) comprise both LPD and MPD. Continental families: LPD 92%, MPD 8%; Faroese family: LPD 70%, MPD 28% (Table 1).

CLL is the most common diagnosis in the continental families (65.6%) compared with a prevalence of 22.8% in the Faroese family. Two of the most common types of malignant lymphoma (FL, follicular lymphoma and DLBCL, diffuse large B-cell lymphoma) have a low prevalence both in the Faroese family (FL 6.4% of all lymphoproliferative disorders, LPD, and DLBCL 15.6% of all LPD) and in the families from Norway and Denmark (FL 6.9% of all LPD, and DLBCL 5.8% of all LPD). Compared with the findings in the national cancer registries in Norway and Denmark (FL 12% and DLBCL 25% of all LPD)^42,43^, it turns out that the prevalence of both types of malignant lymphoma is significantly lower in the family materials (P < 0.05 for both lymphomas).

**Anticipation,** viz. an increased malignancy down through the generations with a lower age of onset of disease, is pronounced in the Faroese family with a high prevalence of ALL (7.3%) compared with 4% in the Cancer Registries. The mean age at onset of disease is lower than in the continental families (P < 0.01). We are uncertain whether the high incidences of HL and MPD in the Faroese family are signs of anticipation.

**Birth order effect** (BOE) which is a rank order by age of the affected sibs in a sib ship, was s only seen in the Continental families, when male CLL patients in paternal lines appear late in the sib ship (P < 0.001).

**Segregation of susceptibility to MBD, Continental families.** Affected parent – affected offspring pairs (P-O pairs) make up the predominant transgenerational components (Table 1). In 138 P-O pairs (LPD 126 and 12 MPD pairs) extracted from the pedigrees, the diagnoses were either CLL or any other diagnosis within MBD, denoted nonCLL (Table 2). The intention of this set up is to expose CLL versus nonCLL in four P-O groups: CLL – CLL, nonCLL – CLL, CLL – nonCLL and nonCLL – nonCLL .

Male AR are predominant in PA, female AR are predominant in MA (P < 0.01). For all four groups comprising all 138 pairs there is a bi-parental affiliation to paternal lines (PA), totally 50 pairs (36%), and to maternal lines, totally 88 pairs (64%) (Table 2). Thus, in CLL- CLL pairs, there is a surplus of affected male offspring in PA, males 12, (71%) and females 5 (29%). In MA, the number of affected CLL female offspring is a little higher than the number of affected males (51%, females versus 49% males).

In pairs with nonCLL offspring (Table 2, sum of total nonCLL children and grandchildren in mixed pairs and in nonCLL—non-CLL pairs the segregation in MA is predominant, where the numer of affected females (21 offspring) is significantly higher than expected (P < 0.05).

All 24 patients with MPD in the familial material (Table 1), mixed with 169 CLL patients of the total 181 familial cases of CLL (Table 1) are included in these pair formations, the rest of the familial patients occur in non-included uncle or aunt – nephew or cousin pairs.

**Segregation of susceptibility to MBD, the Faroese family.** In contrast to the continental families, there are nearly no parent – offspring pairs in the Faroese family and therefore neither sib concordance nor birth order effect (Table 1).

All Faroese ARs to a Pc within 5 generations before Pc were recorded in order to evaluate the parental affiliation of AR related to Pc (Table 3). Hereby, co- and contravariation are visible thanks to the high number of patients of one single family with the reservation that when comparing with the Continental families, the parents are kindred in the Faroese material. Table 3 shows the findings of a systematic study of co- and contravariation of all diagnoses in the Faroese material. Diagnoses not mentioned in Table 3 do not show detectable co- or contravariation. The observed number of patients (OBS) is compared with the calculated, expected number (EXP), cf. Material and Methods, co- and contravariation.

Since a given patient in the generations before Pc may act as AR to several Pcs at the same time, the total number of ARs given in Table 3 is higher than the observed total number of Faroese patients (315 patients) as mentioned in Table 1.

The occurrence of a few affected parent–affected offspring pairs (Table 1) is presumably interference (overlap of multiple pairs of affected non-parent–affected offspring).

**Covariation** concerns AR nonCLL both LPD and MPD (Table 3). Covariation denotes a greater number of observed ARs than expected. The ARs from pairs involved in covariation have a significant correlation to MA (Table 4, P < 0.01, chi-square test, 2 degrees of freedom).

**Contravariation** concerns only AR CLL (Tables 3 and 4). Contravariation means a lower number of observed ARs than expected. The ARs from pairs involved in contravariation have a significant correlation to PA (Table 4, P < 0.05, chi-square test, 2 degrees of freedom).

## Discussion

The findings from the present study on the segregation of susceptibility to MBD (Tables 1, 2, 3, 4), are fused with a model of parental genomic imprinting^22^ (Fig. 1). Parental genomic imprinting denotes a transgenerational segregation that depends on the gender of the parents. One allele is transcriptionally inactivated (imprinted, silenced) while the other allele remains active. At each transgenerational passage, the imprint erases and then it regenerates making imprinting a lifelong mechanism. A distinction between maternal and paternal imprinting points out a stereotypic distribution of affected- and healthy persons, and carriers in the pedigree (Fig. 1).

As shown here, a proband crude (Pc) can have affected relatives (ARs) in paternal lines (PA) with maternal imprinting, and ARs in maternal lines (MA) with paternal imprinting. In this way, there is implicit in parental genomic imprinting basis for specific pathways in the segregation from mother or father to female or male offspring (Fig. 1). We assume that such specific transgenerational pathways are included in the explanation of the differences we have seen for male and female offspring (Table 2).

We have previously argued that the genetic component of MBD may be reappearance of parts of the fetal residual-genome, which long after birth can produce mutated hematopoietic monoclones, as seen in MBD^29^. The regulating factor is a so-called polymorphic equilibrium with segregation distortion related to parental imprinting^23,24^ and mainly to the segregation of genes of early embryonic growth factors^23–25,44^.

In such cases, fitness (in a biological sense) is generally higher than in sheer Mendelian segregation^24^, and the mean fitness is maximized by complete but opposite drive in the two fractions, PA and MA^23,24^. In mammals, fitness usually means maternal drive and maternal predominance, protection of the oocyte, and tight regulation of genes affecting the feto-maternal interface in placenta^45,46^. In CLL^31^ and sometimes in NHL^32,33^, fitness means specifically that the overall 10 years survival and the response to treatments are better in females than in males. From a biological point of view, there is much to suggest that Homo sapiens takes good care of these gene sets for susceptibility to MBD, which master the critically important monoclones of the fetal hematopoiesis. The genes are maintained and "amplified" transgenerationally by anticipation on their way down through the generations, where genes for different diagnoses under MBD are mixed-up by covariation. We have had the opportunity to demonstrate this in a large family, the related Faroese population, with many hundreds of affected people. CLL is an exception that contravariates and thereby "keeps to itself in clusters in the pedigree”, unknown for what reason, perhaps a balance between paternal and maternal inheritance (viz. opposite drive in PA and MA, Table 4). There is also no certain explanation as to why the two lymphomas DLBCL and FL occur with lower frequencies in the families (Table 1) than in the cancer registers^42,43^, we can just state that in the case of inheritance (familial occurrence), a certain sorting of the susceptibility genes seems to takes place. LPD and MPD are found mixed in our family materials (Table 1), which is consistent with the fact that for LPD and MPD, common germ cell mutations have been detected in families with MBD, for example the so-called DDX41 mutation^47^.

Like other (onco-) fetal antigens, the susceptibility genes for monoclonal haematological disease represent a risk of disease later in life after birth. There is an increased risk of leukemias, lymphomas and myeloma and other monoclonal malignant haematological disorders, and also of autoimmune diseases. The mechanisms of inheritance and distribution of the susceptibility genes minimize the consequences of disease. In men with CLL, for example, by birth order effect, where the disease is often seen in sons late in the sibling group, when there are already unaffected healthy older siblings. Another example is the conditions where susceptibility maternally is given to daughters, where with several diagnoses there is a documented better prognosis and a better treatment response to chemotherapy than in men. The vast majority of the clonal malignant blood diseases occur especially after childbearing age, so the presence of the susceptibility genes has little effect on the survival of Homo sapiens. It must be assumed that the benefit of carrying these genes, with their great importance in fetal life, is outweighed by the risk of disease. Evolutionarily, it is undoubtedly an advantage that the MBD susceptibility during inheritance can be managed and distributed with these epigenetic mechanisms, so that this potentially dangerous congenital baggage will not just be given to all descendants according to Mendel's laws.

In general, genomic imprinting of a pluripotent stem cell with subsequent differentiation brings about the same number of affected males and females, carriers and healthy people in PA and MA. The allocation of the stem-cell descendants to either PA or MA is permanent, sometimes denoted «the specific fate”^25^.

If, on the other hand, it is about somatic cell differentiation after the initial pluripotent stage, where newly developed descendants of the stem cell become mother cells for new maturation cell lines, the fate may change^48^. The genes involved can change under the influence of even more polymorphic equilibria, e.g. restricted gene-expression, epigenetic modifies (DNA methylation modifications) and epigenetic reprogramming moderators (clonal development and loss of genomic integrity^25,48^. In following generations, specific monogenes in the segregation of susceptibility to MBD undergo further segregation distortion to fractions of male and female ARs in PA and MA. Our present findings on the strong dominance of male ARs in PA and the modest accumulation of male ARs in MA, can hardly be explained without a polymorphic equilibrium with segregation distortion.

Co- and contravariation of AR are such opposite drives of “the two fractions”, PA and MA. In MA, there is an observed predominance of groups with covariation where PA, however, is not zero. In PA, where groups with contravariation predominate, MA is not zero (Table 4). It might look like a discrepancy that so many female AR CLL from MA contravariate. We believe that AR CLL’s association with PA is correctly reported (Table 4) and that we see an opposite drive between PA and CLL, and between MA and nonCLL.

The physiological HLA-related microchimerism between mother and fetus (transplacental passage of fetal cells to the mother) completes the model of segregation of susceptibility to MBD^49–52^. In that perspective, microchimerism belongs to the group of physiological segregation modifiers. Without the notion of drive of susceptibility by HLA mismatch and chimerism, the model would have no reasonable explanation for birth order effect (per-generational enhancement) or anticipation (trans-generational enhancement) (Fig. 1). Apparently, chimerism between mother and fetus exerts a drive for the susceptibility. We do see affected pairs of mother-son where the HLA mismatch is greatest in families with unrelated parents, but not, or nearly not in families with related parents and thus a higher degree of HLA compatibility (Table 1). Here, MBDs appear more widely distributed in the family tree, presumably at such positions where the susceptibility of the proband can be expressed distant from the father and mother at places with a slightly higher mismatch, for example in the Faroe Islands, where many isolated islands are scattered in the ocean. This is also in accordance with observations from Saudi-Arabia, where consanguineous parents have children with a lower frequency of MBD than seen in families with unrelated parents^53^.

Familial cases of two of the most common subtypes of malignant lymphoma, the diffuse large B-cell lymphoma (DLBCL) and the follicular lymphoma (FL) have been found to have a lower frequency than in solo cases as reported by the Cancer Registries^42,43^.This trend is also seen in the present investigation, but not statistically significant. Hodgkin lymphoma, on the other hand, is well known in familial clustering^54,56^ as also seen in the Faroese family (Table 3) The findings must be taken with the reservation that the patients are alive and without symptoms at the time of registration but they may later in life develop MBD which ought to be included.

### Informed consent

The patients were informed about the purpose of the study, that it was free to decline at any time, and that the investigation was approved by the Scientific Ethical Committees and done according to the Declaration of Helsinki. It was clearly stated that data would remain confidential and unrecognizable outside the study. Each patient confirmed participation by completing a questionnaire, approved by the National Ethical Committees.

## Data Availability

Data are stored at the National Norwegian Cancer Registry, Ullernchausseen 64. NO 0379, Oslo, Norway, att. Dr.Tom Børge Johannesen.
